# Supplementary material for: Recipient pre-existing chronic hypotension is associated with delayed graft function and inferior graft survival in kidney transplantation from elderly donors
Source: PLoS One. 2021 Apr 5;16(4):e0249552. doi: 10.1371/journal.pone.0249552 (PMC8021200; doi:10.1371/journal.pone.0249552)
Supplement: Attachment — Submitted filename: Response to reviewers.docx [file pone.0249552.s003.docx]

*Academic editor*

Many thanks for making the revisions as per the reviewers’ recommendations. They certainly have improved the quality of the MS, but the reviewers still have a few outstanding points that need to be addressed, as i think they are important. They are specified in the comments below. I would encourage you to thoroughly go through them and make the appropriate revisions. I think the MS should be acceptable for publication after that, but will depend on the quality of the revisions and will undergo thorough re-review especially in the light of the outstanding concerns.

*Response. We thank the Academic Editor for his comments. According to reviewers’ suggestion, we re-checked patients schedule for perioperative data (which are now available in 917/1127); as reported below, we found a high concordance between “historical” hypotension and an MBP<80 mmHg immediately before, during (at reperfusion), or after the surgery (K=0.616); however, pre-existing hypotension showed a better association with DGF. As now better expressed in results and discussion sections, our findings suggest that, besides being only related to intraoperative hypotension, chronic pre-existing blood pressure status may better describe a high-risk profile of KTs who may develop DGF.*

*As also indicated by Rev#2, we implemented the multivariate analysis including dialysis type and donor hypertension and confirming that patients in the hypotensive group had the higher risk of experiencing DGF (OR, 5.4; 95% confidence interval [CI], 2.8 to 10.6). As also suggested, the English language has been extensively evaluated.*

*Reviewer #1:* The authors appear to have carefully considered the extensive review comments. the revised version is much clearer and of value to the journal’s readership in highlighting the important consequences of recipient hypotension for kidney transplantation. the manuscript highlights the importance of considering this factor in both policy decisions around allocation and actual management of individual patients. the manuscript makes appropriate suggestions regarding further studies in the area in line with initial reviews.

*Response: We appreciate Rev#1 consideration for our paper.*

*Reviewer #2:*  After the thorough revision the paper is much more focused and has clearly improved. The writing is also clearly improved . I do have one question about the multivariate analysis as shown in table 5. The authors state that the included the significant predictors for DGF as shown in table 2. However the highly significant predictors type of dialysis and donor hypertension are left out. I believe this is not explained in the manuscript. Especially type of dialysis seams an important determinant to ad in this analysis.

*Response: We agree with Rev#2 comments, and, according to his/her suggestions, we implemented the multivariate analysis, including dialysis type and donor hypertension. This final elaboration confirms that patients in the hypotensive group had the higher risk of experiencing DGF (OR, 5.4; 95% confidence interval [CI], 2.8 to 10.6).*

*Reviewer #3:*  Thank you for your revisions and I acknowledge the significant amount of work that the authors have put into the manuscript. However, I have two significant concerns:
1. There remain some significant English grammatical and spelling errors particularly in the introduction, methods and discussion. These would need to be resolved prior to the publication;
2. My previous comments regarding the management in the perioperative period. Having looked at the supplementary data provided, this is insufficient to provide analysis: data are only provided for 89 of the 1127 receipients and it is difficult to draw any meaningful conclusions from the data presented. The outstanding issue of how those patients with pre-existing hypotension were managed in the perioperative still needs to be resolved.
*Response: We thank Rev#3 for his comments. According to his suggestions:*

1- *The English language has been further extensively evaluated*
2- *We re-checked patients schedule for perioperative data (which are now available in 917/1127); as reported, we found a concordance between “historical” hypotension and an MBP<80 mmHg immediately before, during (at reperfusion), or after the surgery (K=0.616); however, pre-existing hypotension showed a better association with DGF [OR for perioperative hypotension 2.8 (1.9-4.1) vs. 5.4 (2.8-10.6)]. As now better expressed in results and discussion sections, our findings suggest that, besides being only related to intraoperative hypotension, chronic pre-existing blood pressure status may better describe a high-risk profile of KTs who may develop DGF. We then stressed in the discussion section the need for a rational peri- and postoperative fluid management to maintain adequate kidney perfusion without a maximization of cardiac filling, considering that a goal-directed therapy may represent the optimal approach in this setting.*
